# Loss and Dysregulation of Th17 Cells during HIV Infection

**DOI:** 10.1155/2013/852418

**Published:** 2013-05-23

**Authors:** Sandra L. Bixler, Joseph J. Mattapallil

**Affiliations:** Department of Microbiology & Immunology, Uniformed Services University of the Health Sciences, Bethesda, MD 20814, USA

## Abstract

Bacterial translocation across the damaged mucosal epithelium has emerged as a major paradigm for chronic immune activation observed during HIV infection. T helper 17 (Th17) cells are a unique lineage of T helper cells that are enriched in mucosal tissues and are thought to play a central role in protecting the integrity of the mucosal barrier and maintaining immune homeostasis at mucosal sites. Th17 cells are lost very early during the course of HIV infection, and their loss has been shown to correlate with bacterial translocation. Interestingly, Th17 cells are unable to completely recover from the early destruction even after successful antiretroviral therapy (ART). Here, we review some of the potential mechanisms for the loss and dysregulation of Th17 cells during HIV infection.

## 1. Introduction

 T helper 17 (Th17) cells have emerged as a key player in host-pathogen interplay at the mucosal surface. The lack of Th17 cells has been associated with recurring bacterial and fungal infections that are a hallmark of hyper-IgE syndrome [1, 2]. Th17 cells are enriched at mucosal sites [3–5] where they are thought to play a role in maintenance of immune homeostasis in response to commensal organisms and protect against pathogens that may gain entry via these surfaces [6]. Studies have shown that a paucity of Th17 cells in mucosal tissues is associated with systemic translocation of bacteria across the intestinal epithelial barrier [7]. 

 Th17 cells are a unique lineage of T helper cells that are induced under anti-Th1/Th2 polarizing conditions and preferentially produce interleukin-17 (IL-17) [8–12] and express markers such as CD26, CD161, and interleukin-4-inducible gene [11, 13–15]. This newly identified subset of Th17 cells was later found to be the key effector T-cell subset mediating experimental autoimmune encephalitis (EAE) in mice [16, 17]. Deletion of Th1 cells was found to exacerbate the symptoms of EAE, and this finally led to identification of Th17 cells as the primary cells mediating the development of EAE [18–20].

IL-17 produced by Th17 cells serves as a chemoattractant for neutrophils to sites of infection and inflammation [21, 22]. IL-17 also promotes tight junction formation at mucosal surfaces through the upregulation of claudin-1, claudin-2, and zona occludens-1 expression, which are all key proteins essential for maintenance of epithelial barrier integrity [23, 24]. Studies have demonstrated that IL-17 increases the production of antimicrobial peptides such as *β*-defensins that play critical roles in defense against microbial pathogens [25–28]. Th17 cells also produce a number of other cytokines such as IL-22 and IL-21 that have been shown to synergize with IL-17 and enhance the expression of antimicrobial peptides in mucosal tissues [26]. Additionally, IL-22 has been demonstrated to be critical for enterocyte homeostasis [29]. Numerous studies have shown that Th17 cells express CCR4, CCR6, CCR9, and *α*4*β*7 [30–33] suggesting that these cells preferentially migrate to mucosal tissues. 

Th17 cells play a critical role in protection against pathogens though they have been implicated in several autoimmune and inflammatory disorders, including asthma and allergy [34], psoriasis [35, 36], and inflammatory bowel disease [37, 38]. Interestingly, recent studies have shown that other cells such as CD8 T cells called T-cytotoxic-17 (Tc17) cells were capable of producing IL-17. Huber et al. [39] showed that IL-17 secretion by CD8 T cells supported Th17-mediated autoimmune encephalomyelitis, whereas Saxena et al. [40] demonstrated that Tc17 cells potentiated Th1-mediated diabetes in the mouse model. Other studies have implicated Tc17 cells in vaccine-mediated immunity against fungal pathogens [41].

## 2. Th17 Cells during HIV Infection

 HIV and SIV infections are characterized by massive loss of T helper cells, particularly at mucosal sites that persists during the course of infection, with little or no repopulation even after long periods of antiretroviral therapy [32, 42–53]. Destruction of mucosal CD4+ T cells is accompanied by dramatic alterations of the mucosal microenvironment, and is characterized by a preferential loss of Th17 cells, intestinal dysfunction and malabsorption, loss of mucosal epithelial barrier integrity, and severe enteropathy [54]. 

The exact mechanisms for the loss of Th17 cells are still under investigation. Brenchley et al. [4] reported that Th17 cells in the mucosa express high levels of CCR5, the coreceptor for HIV, and appear to be preferentially depleted despite the fact that they were not preferentially infected. On the other hand, Hed et al. using phenotypic markers such as CCR6 expression to delineate Th17 cells reported that direct infection by HIV likely played a central role in their depletion [55]. Ndhlovu et al. [56] demonstrated that IL-17 expression was dependent on the extent of infection in HIV-1+ children whereas HIV-infected patients with a plasma viral load below 50 copies/mL had detectable IL-17 expression. Other studies [57] have shown that HIV-1 specific Th17 cells were present in the acute stage of HIV infection yet were undetectable during chronic infection. The exact role that virus-specific Th17 cells play in HIV infection is still under investigation. Interestingly, however, HIV long-term nonprogressors appear to preserve their Th17 subsets [58]. In spite of ongoing debate about the exact mechanisms for the loss of Th17 cells, it is clear from a large number of studies in HIV-infected subjects and nonhuman primates with pathogenic SIV infections that Th17 cells are depleted to some degree during infection and their depletion contributes to the pathogenesis of HIV infection. Recent studies have shown that the Tc17 cells like their counterparts are also depleted during chronic HIV and SIV infections [59, 60].

 In a landmark study, Brenchley et al. [61] showed that HIV infection was accompanied by translocation of microbial products across the lumen of the intestinal mucosa into systemic circulation. These translocated microbial products are believed to be a major cause for chronic immune activation and disease progression characterized by increased cell turnover [61–63]. A number of studies in HIV-infected patients and nonhuman primate models have demonstrated that the loss of Th17 cells from the mucosa most likely plays a major role in microbial translocation. Raffatellu et al. [7] showed that Th17 cell deficiency during SIV infection was associated with systemic translocation of *Salmonella*. Likewise, pathogenic SIV infections are accompanied by a severe loss of Th17 cells at mucosal sites within the first few weeks of infection that persists in chronic infection [64]. In contrast to pathogenic infections, natural hosts of SIV infection such as sooty mangabeys and African green monkeys were found to preserve their Th17 cells following infection and display little or no immune activation even when viral replication is high [4]. 

The effect of HIV and SIV infections on the loss of Th17 cells has been well documented. Not much is, however, known about the ability of Th17 cells to effectively repopulate either during the course of infection or after therapy. Ciccone et al. demonstrated that long-term highly active antiretroviral therapy (HAART) was somewhat successful in achieving Th17 repopulation in both peripheral blood and the mucosa [58]. On the other hand, Macal et al. [65] suggested that Th17 repopulation was dependent on overall levels of CD4+ T cell restoration in the gastrointestinal-associated lymphoid tissue (GALT). Gaardbo et al. [66] reported that ~20% of the HIV patients on antiretroviral therapy failed to completely reconstitute their CD4+ T cells which was accompanied by an incomplete repopulation of Th17 cells. Mavigner et al. [33] showed that incomplete mucosal immune reconstitution was associated with defective gut homing of CCR9+*β*7+ CD4+ T cells, a population that harbored Th17 cells. This was likely due to the altered expression of the CCR9 ligand CCL25 in the small intestinal mucosa of HIV-infected individuals. He et al. [67] reported that HIV-infected patients had significantly low levels of Th17 cells that were partially restored after 6 months of HAART though higher levels were observed after 1 year of therapy. Likewise, elite control of HIV infection has been associated with higher levels of Th17 cells [68]. However, others have demonstrated that HAART failed to restore Th17 cells in HIV-infected patients undergoing therapy [55, 69]. The inability to effectively repopulate Th17 cells unlike other subsets such as Th1 or Tregs suggests that mechanisms that likely affect either the induction or differentiation of Th17 cells may be involved in the poor repopulation of Th17 cells.

Even though HAART has had limited effect on Th17 repopulation, recent studies suggest that using probiotics can potentially enhance gastrointestinal immunity, enhance CD4+ T cell numbers, and lead to the restoration of Th17 cells in the mucosa [70]. Klatt et al. [71] showed that treatment of SIV infected pigtail macaques with probiotics/prebiotics for 60 days along with antiretroviral therapy was accompanied by an increase in IL-23 producing cells and higher levels of multifunctional Th17 cells in the mucosa as compared to animals that only received antiretroviral therapy. Likewise, Gonzalez-Hernandez et al. [72] showed that symbiotic treatment of HIV-infected subjects with a combination of pre- and probiotics significantly decreased microbial translocation and inflammation and improved the immunological status of patients leading to a better long-term outcome. However, another randomized clinical trial [73] reported no major changes in either microbial translocation or markers of immune activation. It is not clear if a better outcome would have been observed with either longer periods of symbiotic treatment or if patients were on antiretroviral therapy at the time of symbiotic therapy. Additional studies are needed to assess the beneficial role of symbiotic therapy on Th17 reconstitution.

## 3. Regulation of Th17 Cells and HIV Infection

 Like the other T helper subsets, Th17 cells are memory CD4+ T cells [30, 69] that differentiate from naïve CD4+ T cells after TCR stimulation and costimulation by antigen presenting cells (APC) in the presence of Th17 promoting cytokines [74–76]. 

 Development of Th17 cells requires key cytokine signals, several of which are produced by APCs following activation of toll-like receptors (TLRs) by pathogen-associated motifs. Activation of TLR 1/2, TLR 3, TLR 4, TLR7/8, and TLR9 have been shown to promote development of Th17 cells [74–78]. Fukata et al. [79] also demonstrated a role for MyD88 induction in Th17 differentiation. Reynolds et al. [80] showed that Th17 cells express high levels of TLR2 and stimulation with TLR2 agonists in the presence of Th17-promoting cytokines led to increased IL-17 production and expression of Th17-associated gene products. Signaling through other molecules such as dectin-1 and DC-SIGN has also been shown to promote Th17 development [81–85]. 

 Initial studies identified IL-6, IL-21, IL-23, and TGF*β* as critical cytokines essential for the induction of Th17 cells. A number of studies using mouse models suggested that IL-6 and TGF*β* were essential for the initial differentiation of Th17 cells. Unlike mouse, however, the studies in humans have suggested that any of the four cytokines along with IL-1*β* in different combinations were sufficient to induce Th17 cells [85–87]. Of the four Th17 promoting cytokines, IL-6 and TGF*β* appear to be critical for the polarization of Th17 cells as Th17 cells produce IL-17 and IFN*γ* in the absence of TGF*β*.

IL-6 binding to the IL-6 receptor initiates signaling through STAT3 and ROR*γ*t transcription factors leading to the STAT3-mediated activation of the IL-17 promoter and the induction of IL-21 and IL-23 receptor expression, two factors that are important for subsequent stages of Th17 development [88]. The essential requirement of IL-6 for the generation of Th17 cells came from studies showing that expression of mutant gp130 IL-6R [89] or treatment with an anti-IL-6 antibody prevented Th17 polarization [76, 90]. 

 Unlike IL-6, the ability of TGF*β* to polarize Th17 cells appears to be dependent on the concentration of TGF*β* in the environment; low concentrations of TGF*β* in the presence of other Th17-promoting cytokines drives ROR*γ*t expression and induces Th17 cells. On the other hand, high concentrations of TGF*β* in the absence of other Th17 inducing cytokines promote the development of T regulatory (Treg) cells and inhibit Th17 development through an effect on the Treg transcription factor FoxP3. TGF*β*1 deficient mice have low levels of Th17 cells and circulating IL-17 [91] whereas treatment with anti-TGF*β*1 antibodies were found to inhibit the generation of Th17 cells [92].

 The second stage of Th17 differentiation is mediated by IL-21, a member of the common gamma chain family of cytokines. IL-21 is an autocrine cytokine that provides a positive feedback mechanism for the induction of Th17 cells [93, 94] and has been shown to inhibit FoxP3, thereby skewing the development away from Tregs. IL-21 has been shown to promote the induction of IL-17 and block IFN*γ* production [93–96] whereas other studies have shown that IL-21 knockout mice or IL-21R-deficient mice fail to develop Th17 cells when stimulated with IL-6 [93–97]. Interestingly, one study reported that IL-21 can subvert the requirement for IL-6-mediated stimulation for inducing Th17 cells by promoting an alternative pathway; a combination of IL-21 and TGF*β* was found to induce Th17 cells in IL-6 deficient mice [98]. 

 Like IL-21, IL-23 appears to be critical for the differentiation of Th17 cells during later stages of development. IL-23 is a heterodimeric cytokine comprised of the IL-12p40 and p19 subunits that is induced by stimulation of dendritic cells and macrophages with different TLR2 and dectin-1 ligands [84, 85, 99]. IL-23 binds to the IL-23 receptor which is primarily expressed by activated memory T cells [100]. Initial studies suggested that IL-23 was essential for the Th17 polarization. Later studies, however, demonstrated that it was not required for initial differentiation of Th17 cells but was essential for the survival and expansion of Th17 cells [8, 9, 101]. Importantly, naïve CD4+ T cells were found to lack the IL-23 receptor. This further supports a role for IL-23 in the later stages of Th17 differentiation.

Interestingly, both HIV and SIV infections are characterized by high levels of IL-6 and TGF*β* [102–104]. Conversely, IL-21 producing CD4+ T cells are lost very early in infection [105–107] though other cellular subsets such as CD8 T cells have been shown to upregulate IL-21 production [107–111]. The presence of high levels of Th17 promoting cytokines during HIV and SIV infections suggests that the failure to induce Th17 cells during infection is likely mediated by mechanisms unrelated to availability of these cytokines. 

Recent studies have shown that the loss of Th17 cells was accompanied by an expansion of Treg cells. These studies have suggested that the accumulation of byproducts of tryptophan metabolism promotes the development of Treg's and inhibits Th17 cells. Indoleamine deoxygenase (IDO), a rate-limiting enzyme that mediates the catabolism of tryptophan, has been shown to be significantly upregulated during HIV and SIV infections [68, 112–115]. Likewise the frequency of Tregs was reported to be altered during HIV infection and during HAART [116–120] whereas effector IL-17 absolute cell numbers were significantly lower in all HIV(+) subjects tested and were not restored after therapy. On the other hand, Brandt et al. [68] showed that the ratio of Th17/Treg in elite controllers did not differ from that of uninfected controls, whereas the ratio was lower in viremic patients and patients on HAART.

It is not clear if HIV infection alters the signaling pathways that promote the induction of Th17 cells. ROR*γ*t is a lineage specific transcription factor associated with Th17 differentiation [88, 121, 122] whose expression is regulated by signal transducers and activators of transcription-3 (STAT3) [123, 124]. They bind to ROR-dependent enhancer elements in conserved noncoding sequence (CNS)-2, which is located upstream of the IL17A promoter [124]. Rueda et al. [125] examined expression of T helper lineage-specific transcription factors in the GALT from healthy uninfected volunteers, HIV-infected untreated, and patients undergoing HAART and found that the ratio of ROR*γ*t to FoxP3 expression shifted in favor of FoxP3 in untreated patients, though ROR*γ*t expression itself was not changed among the groups. 

Numerous studies have demonstrated the importance of the Janus-associated kinases (JAK)/STAT3 signaling pathway in the development of Th17 cells [126, 127]. Binding of Th17 promoting cytokines to their cognate receptors initiates the signaling cascade that leads to receptor dimerization and recruitment of JAK culminating in the activation and phosphorylation of STAT3. Activated pSTAT3 dimerizes and translocates to the nucleus where it binds to the IL-17 promoter and drives the induction of IL-17. Studies have shown that STAT3 knockout mice failed to develop Th17 cells [123, 128], whereas patients with Jobs' syndrome lack functional STAT3 and display impaired Th17 development [2].

STAT3 is negatively regulated by a number of factors such as the suppressor of cytokine signaling-3 (SOCS3), protein inhibitor of activated STAT3 (PIAS3), and protein tyrosine phosphatase (SHP-2). Overexpression of SOCS3 has been shown to inhibit Th17 development while SOCS3 conditional knockouts were shown to have higher levels of Th17 cells [129]. Interestingly while SOCS3 is activated by Th17 promoting cytokines IL-6, IL-21, and IL-23 [92, 129], TGF*β* has been shown to inhibit SOCS3 induction by IL-6 and IL-23, thereby promoting the activation of STAT3 and subsequent induction of Th17 cells [92]. 

CD4+ T cells from HIV-infected patients have been shown to express high levels of SOCS3 mRNA [130] though SOCS3 protein levels were lower. Higher levels of SOCS3 mRNA have been reported in the gastrointestinal tissues of SIV-infected rhesus macaques [131]. Interestingly, increased levels of SOC3 have been shown to aid in HIV replication [132] whereas high levels of SOCS3 in hepatic cells have been associated with nonresponsiveness to therapy in HIV/HCV infected individuals [133]. Moutsopoulos et al. [134] reported that high levels of SOCS3 protein in mucosa-associated lymphoid organ such as the tonsils are associated with increased susceptibility to HIV infection.

Unlike SOCS3, PIAS3 has been shown to directly interact with pSTAT3 and inhibit its binding to target DNA thereby interfering with the STAT3-mediated activation of target genes [135, 136]. Others have shown that PIAS3 directly inhibits the transactivation of STAT3 [137]. PIAS3 transcript levels were found to be absent in Th17 cells as compared to Th1 or Th2 cells in mice, and knockdown of PIAS3 with siRNA resulted in severe EAE suggesting an important role for PIAS3 in Th17 regulation [138]. Recent studies have shown that PIAS3 mRNA levels were significantly upregulated in CD4 T cells from SIV-infected rhesus macaques and high levels of PIAS3 was found to significantly correlate with immune activation and markers of microbial translocation [139]. Not much is known about the effect of HIV infection on PIAS3 and if PIAS3 plays a role in dysregulating the induction of Th17 cells.

Like SOCS3 and PIAS3, SHP2 negatively regulates IL-17 production. However, unlike the other two, SHP2 interferes with IL-6 signaling-mediated activation of STAT3. SHP2 is recruited to receptors following cytokine signaling and JAK activation. Studies have shown that SHP2 is recruited to gp130 domain of the IL6 receptor following IL-6 signaling and dephosphorylates pSTAT3, preventing its dimerization and translocation to the nucleus [140, 141]. The exact role of SHP2 in preventing the induction of Th17 cells during HIV infection is not clear. However, studies have shown that HIV-mediated signaling through CCR5 and C-type lectin domain-4 (DCIR) results in recruitment of SHP-2 whereas HIV gp120 binding has been shown to increase SHP2-mediated signaling [142].

## 4. Summary

 Th17 cells play an essential role in host immunity and are key players in protecting the mucosal integrity. Their loss during HIV infection is associated with translocation of microbial products across the damaged mucosal epithelium leading to immune activation and poor long-term outcome in HIV-infected patients. While progress has been made in understanding the role of Th17 cells in HIV infection, there are significant gaps in the field regarding the exact mechanisms that prevent full Th17 reconstitution during therapy. A better understanding of how these key molecular mechanisms are altered during HIV infection and the role these altered mechanisms play is essential to develop better therapeutic approaches to repopulate Th17 cells and overcome the deleterious effects associated with the loss of Th17 cells during HIV infection.
